# Combined transcriptomics and proteomics forecast analysis for potential biomarker in the acute phase of temporal lobe epilepsy

**DOI:** 10.3389/fnins.2023.1145805

**Published:** 2023-03-30

**Authors:** Cong Huang, Zhipeng You, Yijie He, Jiran Li, Yang Liu, Chunyan Peng, Zhixiong Liu, Xingan Liu, Jiahang Sun

**Affiliations:** ^1^Department of Neurosurgery, The Second Affiliated Hospital of Harbin Medical University, Harbin, China; ^2^Department of Neurology, The Second Affiliated Hospital of Harbin Medical University, Harbin, China; ^3^Department of Orthopedics, Xinyu People’s Hospital, Xinyu, China

**Keywords:** temporal lobe epilepsy, TMT/iTRAQ labeling quantitative proteomics, transcriptomics, biomarker, LASSO, SVM-RFE

## Abstract

**Background:**

Temporal lobe epilepsy (TLE) is a common chronic episodic illness of the nervous system. However, the precise mechanisms of dysfunction and diagnostic biomarkers in the acute phase of TLE are uncertain and hard to diagnose. Thus, we intended to qualify potential biomarkers in the acute phase of TLE for clinical diagnostics and therapeutic purposes.

**Methods:**

An intra-hippocampal injection of kainic acid was used to induce an epileptic model in mice. First, with a TMT/iTRAQ quantitative labeling proteomics approach, we screened for differentially expressed proteins (DEPs) in the acute phase of TLE. Then, differentially expressed genes (DEGs) in the acute phase of TLE were identified by linear modeling on microarray data (limma) and weighted gene co-expression network analysis (WGCNA) using the publicly available microarray dataset GSE88992. Co-expressed genes (proteins) in the acute phase of TLE were identified by overlap analysis of DEPs and DEGs. The least absolute shrinkage and selection operator (LASSO) regression and support vector machine recursive feature elimination (SVM-RFE) algorithms were used to screen Hub genes in the acute phase of TLE, and logistic regression algorithms were applied to develop a novel diagnostic model for the acute phase of TLE, and the sensitivity of the diagnostic model was validated using receiver operating characteristic (ROC) curves.

**Results:**

We screened a total of 10 co-expressed genes (proteins) from TLE-associated DEGs and DEPs utilizing proteomic and transcriptome analysis. LASSO and SVM-RFE algorithms for machine learning were applied to identify three Hub genes: Ctla2a, Hapln2, and Pecam1. A logistic regression algorithm was applied to establish and validate a novel diagnostic model for the acute phase of TLE based on three Hub genes in the publicly accessible datasets GSE88992, GSE49030, and GSE79129.

**Conclusion:**

Our study establishes a reliable model for screening and diagnosing the acute phase of TLE that provides a theoretical basis for adding diagnostic biomarkers for TLE acute phase genes.

## 1. Introduction

Temporal lobe epilepsy (TLE) is characterized by repeated spontaneous seizures, cognitive impairment, and depression which account for 36% of refractory epilepsy cases (Blumcke et al., 2017). TLE has a diverse variety of etiologies, a multitude of clinical features and epigenetics, and high nitrogen, and it is difficult to diagnose and treat (Rawat et al., 2020). EEG is presently used in conjunction with good clinical and historical data to diagnose the acute phase of TLE. Nevertheless, it might be problematic to establish the diagnosis when symptomatic and history characteristics are not typical (Krumholz et al., 2015). Consequently, a currently critical need is to identify biomarkers related to the acute phase of TLE for early diagnosis and therapy.

Diagnostic molecular markers related to changes in biological response can be utilized to identify the development of epileptic disease (Banote et al., 2022). For instance, levels of S100 calcium-binding protein B (S100B) (Liang et al., 2019; Simani et al., 2020), Glial fibrillary acidic protein (GFAP) (Simani et al., 2018), and Ubiquitin carboxy-terminal hydrolase L1 (UCHL-1) (Mondello et al., 2012) can be detected in the blood of epileptic patients as potential biomarkers. Inflammation is regarded to be an important process in epileptogenesis (Vezzani et al., 2016), which is why neuroinflammatory indicators may also be potential epilepsy biomarkers.

Bioinformatics and microarray technologies are frequently used to examine genetic changes at the transcriptional regulation and to discover and annotate differentially expressed genes (DGEs) (Li et al., 2017). Proteomics has already gained a great deal of attention for the search for new biomarkers that deepen our understanding of the molecular basis of disease (Sarkis et al., 2017). Quantitative proteome, as opposed to genomes and transcriptomics, is the preferred approach to a thorough knowledge of overall proteome differences. In the realm of epilepsy, the use of TMT/iTRAQ labeling and bioinformatics analysis can elucidate potential biomarkers and mechanisms through proteomics (Sun et al., 2020). Analysis based on the combination of proteomics and transcriptomics can take full advantage of the differences and complementarities between transcriptomic and proteomic studies to measure the full spectrum of gene expression levels to discover new molecules that conventional single-omics studies fail to discover. Several scholars have identified possible molecular mechanisms of accidents and sudden death in epilepsy by combined proteomics and transcriptomics analysis (Leitner et al., 2021). WGCNA (weighted gene co-expression network analysis) has regarded as the most widely used gene screening instrument. They were demonstrated *via* constructing free proportion genes coexpression nets under a variety of conditions to investigate the link between clinical manifestations and coexpression pattern genes. Furthermore, numerous methodologies for machine learning are being increasingly adopted in the medical field. The LASSO and SVM-RFE algorithms have been proven to be helpful in biomedical uses. Certain authors used LASSO and SVM-RFE linkage analysis to discover potential ferroptosis biomarkers in coronary artery disease (Wu et al., 2022) and to validate diagnostic models for pulmonary hypertension (Xu et al., 2022). The utilization of the WGCNA plus machine learning to examine data on epilepsy pathophysiology to discover epilepsy vulnerability genes may be ground-breaking.

**FIGURE 1:**
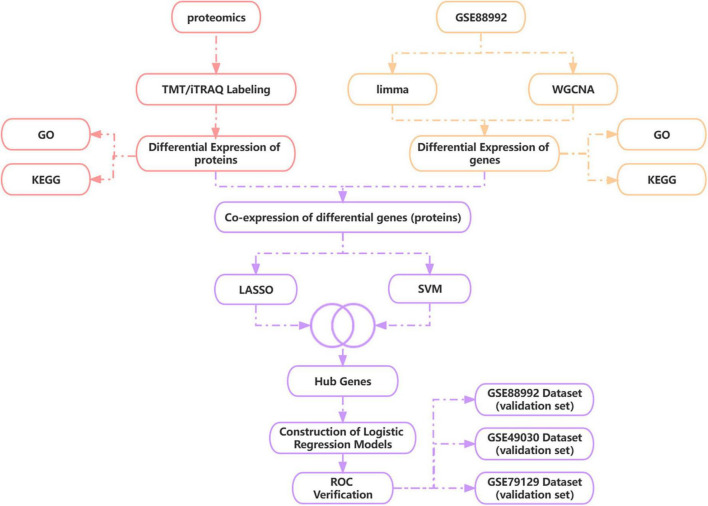
Technology route. WGCNA, weighted gene co-expression network analysis; LASSO, least absolute shrinkage and selection operator; SVM-RFE, support vector machine recursive feature elimination.

During this research, we obtained epilepsy brain tissue-source datasets throughout the GEO database and utilized WGCNA plus analysis of a differential expression to sieve DEGs from them in the acute phase of TLE versus normal control samples. We used TMT/iTRAQ quantitative labeling proteome study to obtain DEGs inside the acute phase of TLE versus normal controls and then combined proteomic and transcriptomic analysis to discover differentially co-expressed genes (proteins) and screen for Hub genes using LASSO and SVM-RFE algorithms. Based on the Hub gene, a novel diagnostic model for the acute phase of TLE was generated using a logistic regression algorithm, and the precision of the diagnostic model was validated using three independent data sets, providing a new perspective for TLE early diagnosis and prevention. Figure 1 depicts the technological pathway.

## 2. Materials and methods

### 2.1. Animals

Male C57BL/6J mice, 8–10 weeks of age, weight 18–20 g, were purchased from the Animal Laboratory of the Second Affiliated Hospital of Harbin Medical University (Harbin, China). Mice were housed in individual cages at 50–60% humidity and we maintained a 12-h light/dark cycle, feeding, and watering each mouse *ad libitum*. Mice fasted for 12 h before surgery. The experimental procedure was carried out in strict accordance with the relevant regulations of the Ethics Committee of the Second Affiliated Hospital of Harbin Medical University (Ethical approval No. SYDWGZR 2020-229; Harbin, Heilongjiang, China). A total of 6 mice were divided into the control group (*n* = 3) and the epilepsy group (*n* = 3).

### 2.2. The model of kainic acid-induced epilepsy in mice

Intrahippocampal injections of hippocampal kainic acid generated epileptic mice with pathophysiology similar to those seen in individuals with temporal lobe epilepsy. Intraperitoneal doses of 0.01 mL/g 1% sodium pentobarbital liquid were performed to anesthetize mice during surgery. In preparation to stereotactically plant a 1 μL microinjector into the right hippocampus region during intra-hippocampal injection, the relevant locations utilized: anterior-posterior (AP) = −2.7 mm, medial-lateral (ML) = −1.8 mm, and dorsal-ventral (DV) = −2.1 mm. Within 2 min, kainic acid (0.4 μg, mixed in 0.8 μL saline; Sigma, St. Louis, MO, USA) was administered (Krook-Magnuson et al., 2013). For 10 min the syringe was left in position to prevent reflux along the administration trajectory. Pseudo-operated mice were administered 0.8 μL saline. The Racine scale (Racine, 1972) was adopted to evaluate seizure severity; mice having seizures more so than or equivalent to Racine IV were deemed positive for successful status epilepticus (SE). To reduce as far as practicable the loss of mice caused by the persistent status epilepticus (>30 min of Racine III-V seizures), diazepam (7.5 mg/kg) was administered intraperitoneally 1 h after the first Racine V seizure. Saline-injected mice (sham-operated controls) also accept intraperitoneal injections of diazepam. These mice were included in the follow-up research. Control and epileptic mice were killed by using the cervical dislocation method on the third day after SE, and the right hippocampus tissue was extracted. Before being handled in proteomics-related investigations, these samples were kept at −80°C.

### 2.3. Proteomics section

#### 2.3.1. Protein extraction and tryptic enzyme digestion

The hippocampus tissue was withdrawn from −80°C, weighed, and placed in a pre-cooled mortar with liquid nitrogen, where liquid nitrogen was added until the hippocampal tissue was powdered. Every batch of the sample was given four times the powder volume of lysis buffer (8 M urea, 1% protease inhibitor, and 1% phosphatase inhibitor), which could then be used to lyse the samples using ultrasound. Cell debris was removed by centrifugation at 12,000 *g* for 10 min at 4°C. The supernatant was transferred to a new centrifuge tube and the protein concentration was determined using a BCA kit (Beyotime Biotechnology, Shanghai, China). Under the circumstance of avoiding light, the obtained proteins were reduced by 5 mM dithiothreitol at 56°C for 30 min next and alkylated by 11 mM iodoacetamide at 25°C for 15 min. Then, 100 mM ammonium bicarbonate was an addition to the solutions obtained above, and these solutions were diluted to a final urea concentration <2 M. Finally, parenzyme (Promega, Madison, WI, USA) was administered (1:50 parenzyme-protein quantity ratio), and the proteins were digested up overnight at 37°C and again for 4 h with parenzyme (parenzyme-protein amount ratio of 1:100).

#### 2.3.2. TMT/iTRAQ labeling and HPLC fractionation

The solution was desalinated using a Strata X C18 SPE column (Phenomenex, Torrance, CA, Canada) and subsequently vacuum centrifuged for dehydration. Protein peptides were dissolved using 0.5 M triethylammonium formate (Sigma, St. Louis, MO, USA) and marked following the TMT/iTRAQ kit operating manual (Thermo Fisher Scientific, Carlsbad, CA, Canada). Peptide mixes marked with TMT/iTRAQ were dissolved and subsequently classified utilizing the C18 column in high pH inverted HPLC (Agilent 300Extend C18 4.6 × 250 mm, 5 μm, Agilent, Santa Clara, CA, Canada). In brief, to simplify, the peptides are subjected to a 60-min gradient of 8–32% acetonitrile (pH 9.0), after which they are separated into 60 fractions. Finally, the 60 fractions are lyophilized after being integrated into 18 fractions.

#### 2.3.3. Liquid chromatograph mass spectrometer (LC-MS) analysis

The TMT/iTRAQ quantified tagging proteome analysis improves on the conventional technique in several ways (Yang et al., 2020). The quantifying proteomics analysis system is composed of two operational systems, the EASY-nLC 1,000 UPLC system and the Q Exactive™Plus Orbitrap MS system (Thermo Scientific, Waltham, MA, USA). Thermo EASY-Spray™ C18 LC column (2 μm, 100 A, 75 μm × 50 cm, Thermo Scientific, Waltham, MA, USA) is to be operated at 40°C. The mobile phase was assembled of 90% (v/v) aqueous acetonitrile, where solvent A was utilized to dissolve the tryptic peptide, and 2% (v/v) aqueous acetonitrile (solvent A), both of which included 0.1% (v/v) formic acid. The 60-min class wash was operated as described in the following: 0 min, 9% B; 38 min, 26% B; 52 min, 35% B; 56 min, 80% B; and 60 min, 80% B. Throughout the experiment, the flow was maintained at 500 nL/min. The peptides from the preceding handling steps were separated with an ultra-performance liquid chromatography system, which was pushed into an NSI ion source for ionization and then subjected to analysis using an Orbitrap Fusion Lumos mass spectrometer. Each peptide’s mother ion as well as its secondary fragmentation were identified and examined utilizing high-resolution Orbitrap, with the ion source energy adjusted at 2.4 kV. The first mass spectrometry scanned range was adjusted to 350–1,550 m/z, with a scan precision of 60,000, while the secondary mass spectrometry scanned range was adjusted to 100 m/z, with a precision of 15,000. Its data processing form employs a data-dependent archiving (DDA) procedure, in which 20 parent peptide ions with both the greatest signal intensities are sequentially chosen to enter the HCD crash cell for separation utilizing 32% of the segmentation power generation that after the primary scan and the same sequential secondary mass spectrometry analysis is carried out. To enhance the efficient exploitation of the mass spectrum, the automatic gain control (AGC) was set up to 5E4, the signaling limit was set up to 10,000 ions/s, the largest input time was set up to 60 ms, as well as the mobile elimination time of the tandem mass spectrometry scan was set up to 30 s to prevent the repetition of the mother ion scan.

#### 2.3.4. Database search

The approach originally provided is the foundation for the data processing, with certain modifications (Wang et al., 2022). The MaxQuant (v2.2.0.0)^1^ analysis was applied to process the mass spectrometry data. Search input parameters included a database for Mus musculus (16,992 sequences), an inverted library to assess the false positive rate (FPR) owing to randomized matching, and general contamination libraries to remove the influence of interfering proteins in the recognition findings. The digestion mode was designated to Trypsin/P, the amount of missed cut sites were assigned to two, the minimum peptide length was assigned to seven residues of amino acids, the maximum of peptide modifications was assigned to five, the mass error tolerance of the primary mother ion was assigned to 20 and 5 ppm for the first and main searches, respectively, and the mass error tolerance of the second fragment ion was assigned to 0.02 Da. The constant modification was cysteine alkylation, while the changeable modifications were methionine oxidation, acetylation, and deamidation (NQ) of the protein’s N-terminus. For both protein identification and PSM identification, the quantification technique was adjusted to TMT-6plex, and the FDR was fixed at 1%.

#### 2.3.5. Differential protein analysis

Protein profile data were identified and variations in protein expression were evaluated utilizing multifactorial statistical analysis, including the principal component analysis (PCA) as well as the Pearson correlation coefficient (PCC) using the R package. In addition, the threshold for DEP was set at a Fold Change (FC) > 1.20 for upregulation and FC < 0.83 for downregulation, and *p*-value < 0.05. The pheatmap package (version 1.0.12) was used to heat-treat DEPs, and ggplot2 package (version 3.3.5)’s volcano mapping was used to visualize differentially expressed proteins.

### 2.4. Transcriptomics section

#### 2.4.1. Download and processing of expression spectrum data

The datasets GSE88992, GSE49030, and GSE79129 regarding epilepsy profiles of gene expression used during this investigation were downloaded from the GEO data set (Table 1).^2^ The soft annotation list of the relevant platform is where microarray probe annotation information is available. A Perl language script (v5.36.0)^3^ will be used to annotate genes. When numerous probes that shared the same gene symbol were found during data annotation, we determined the gene expression level utilizing the average probe expression.

**TABLE 1:** Information derived from the GEO database.

Location	ID	Probe platform	Number
Hippocampus	GSE88992	GPL1261	9 control vs. 8 epilepsy
Hippocampus	GSE49030	GPL1261	9 control vs. 15 epilepsy
Hippocampus	GSE79129	GPL6887	3 control vs. 3 epilepsy

#### 2.4.2. Differential expression analysis

Differential expression analysis was performed on the gene expression profiling dataset GSE88992 between Epilepsy and control using the limma package (version 3.48.3) for R language (Ritchie et al., 2015). The criterion for the significance of DEGs was set up as Fold Change > 1.5 and corrected *p*-value < 0.05. The pheatmap package (version 1.0.12) and the ggplot2 package (version 3.3.6) were used to construct heat maps and volcano plots to visualize the DEGs, respectively.

#### 2.4.3. Establishing a weighted gene co-expression analysis network

The expression profile matrix was first read in R, and the median absolute deviation (MAD value) of each gene was determined individually, with the top 50% of genes with the least MAD values being eliminated. The goodSampleGenes package in R language was applied to eliminate outlier genes and samples, as well as to further build a scale-free co-expression network. The modules were subsequently identified using a hierarchical clustering of genes. Pearson’s correlation analysis was used to assess the relationship between clinical characteristics and the module acquired. Following the criteria of gene significance (GS) > 0.1 and module membership (MM) > 0.8, we chose the essential module with the greatest link with control and Epilepsy as the subsequent step in the analysis.

#### 2.4.4. Genes associated with weighted co-expression networks and differentially expressed genes overlap

Differential expression analysis was obtained for 1,800 differentially expressed genes overlapping with WGCNA-related module genes. The results were visualized using Venn diagrams (Bardou et al., 2014).

#### 2.4.5. Functional enrichment analysis

To reflect the biological function of 1,168 DEGs (692 up-regulated DEGs, 476 down-regulated DEGs) and 140 DEPs (95 up-regulated DEPs, 45 down-regulated DEPs), the clusterProfiler package (version 3.14.3) of R was used for enrichment analysis, and the background sets for GO annotation were the gene sets from the org.Mm.eg.db package (version 3.1.0). The genes were mapped to the background sets, and the results of the enrichment analysis were obtained using the clusterProfiler package. The genes were mapped to the background set and enriched using the R package clusterProfiler (version 3.14.3) for enrichment analysis to obtain enrichment results. The background set was the most recent gene annotation set for KEGG Pathway, which was downloaded from the KEGG rest API.^4^ The minimum and maximum gene collections were designated at 5 and 5,000, respectively, and a *P*-value of < 0.05 was deemed as statistically meaningful.

### 2.5. Screening on co-expressed differential genes (proteins)

Co-expression of differential genes (proteins) was screened by overlaying the 1,168 differential genes obtained from the above gene overlap with 140 differentially expressed proteins. The results were visualized using Venn diagrams.

### 2.6. Screening of Hub genes and construction and validation of diagnostic models

Least absolute shrinkage and selection operator and Support vector machine recursive feature elimination algorithms were applied to screen Hub genes predicated on the above transcriptome and proteomic co-expression of differential genes (proteins). LASSO is a statistical linear regression analysis algorithm that uses regularization to enhance regression results’ prediction accuracy. The punishment variable (λ) of the LASSO regression model was cross-validated ten times by using the λ values corresponding to the lowest partial likelihood deviation. The R package “glmnet” (Friedman et al., 2010) was applied to run the LASSO algorithm to seek genes associated with epilepsy. Furthermore, SVM-RFE is a sophisticated features extraction strategy that finds the optimal factors by eliminating the feature vectors created by SVM (Wang and Liu, 2015). The SVM-RFE algorithm shortlisted the best variables for our investigation based on a minimal 10 CV error value. In our research, we consolidated the genes found by the SVM-RFE and LASSO regression algorithms to identify Hub genes. Supported by the 3 Hub genes, a novel diagnostic model of epilepsy was developed using a logistic regression algorithm. The precision of our previously constructed Hub gene and novel diagnostic model was appraised by the region under the curve (AUC). Therefore, we calculated the AUC values of the three Hub genes and the logistic regression model separately to evaluate the accuracy of the diagnostic model. In the end, the qROC package (version 1.18.0) and the ggplot2 package (version 3.3.5) in R were used, respectively, to compute the region under the AUC curve and plot the curve. We used three independent datasets, GSE88992, GSE49030, and GSE79129, to validate the accuracy of the Hub gene as a diagnostic molecule.

## 3. Results

### 3.1. General information on mass spectrometry

**FIGURE 2:**
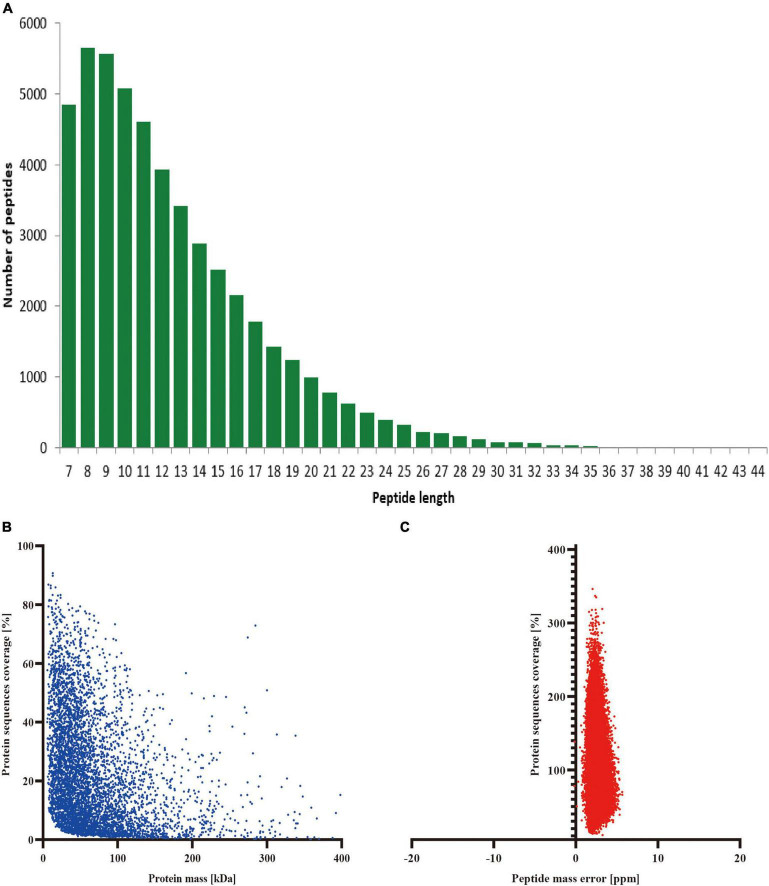
Stability evaluation of mass spectrometry identification. **(A)** Length distribution of peptides identified by mass spectrometry; **(B)** Molecular weight and sequence overlap of the identified proteins; **(C)** Mean peptide mass error.

The precise identification and measurement of the protein peptides are major determinants of the mass spectrometer analysis’s accuracy. As can be noticed from our results, most of the protein-peptide sequences in our proteomics part of the experiments were distributed at 7–25 amino acids, which results are very well in line with the general regulations of trypsin digestion and HCD segmentation (Figure 2A). We can infer from this finding that the length of the protein peptides also complies with the standards for quality control because the proteins isolated from cerebral tissue were found to have high sequence overlap and that the molecular weight of the proteins was negatively correlated with the overlap (Figure 2B). In addition, the quality error spread of the mass spectra tends to be close to zero, with most of the quality errors <10 ppm (normal <70 ppm), which guarantees the high-precision demand of the mass spectra (Figure 2C). All of the aforementioned findings show that the mass spectrometer we utilized is of normal accuracy and has no negative effects on the qualitative and quantitative analysis of proteins because of difficulties with mass spectrometry accuracy.

### 3.2. Protein quantification and analysis of protein expression profile differences

**FIGURE 3:**
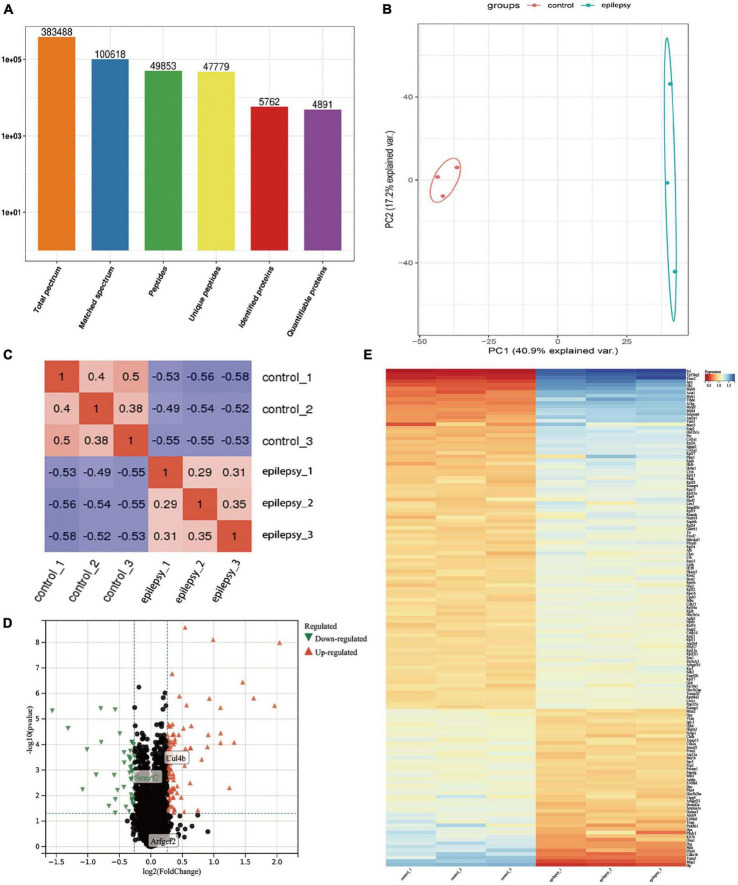
Protein identification information and multivariate statistical analysis based on proteomics analysis. **(A)** Basic statistics of mass spectrometry data results; **(B)** PCA score plots of hippocampal tissue samples between epilepsy and control groups; **(C)** Pearson correlation coefficients of hippocampal tissue samples between epilepsy and control groups; **(D)** Volcano plots of hippocampal tissue samples between epilepsy and control groups, where *x*-axis represents log_2_ (fold change) and *y*-axis represents –log_10_ (*p*-value). Green triangles represent down-regulated genes, red triangles represent up-regulated genes, and black dots represent proteins with no significant differential expression; **(E)** Heatmap of differentially expressed proteins. Each column in the graph represents a sample, each row represents a gene, and the expression status of genes from low to high is indicated by red to blue, respectively.

Under 1% FDR, our protein peptides were identified from a total of 5,762 proteins, of which 4,891 proteins could be quantified (Figure 3A). Next, PCA and PCC were performed on 4,891 proteins. The results demonstrated that the PCA core graphs were significantly separated at 95% confidence intervals (Figure 3B), and there was a significant distinction between the two groups in the PCC graphs (Figure 3C). The above results all demonstrated that the differences in protein expression profiles between epilepsy and control groups were statistically significant and fully satisfied the criterion and could be used for subsequent analysis. Finally, we obtained 140 DEPs, which included 95 DEPs remarkably upregulated and 45 DEPs remarkably downregulated (Supplementary Data Sheet 1). The results of DEPs are illustrated in the volcano plot (Figure 3D) and heat map (Figure 3E).

### 3.3. Transcriptome differential expression analysis

**FIGURE 4:**
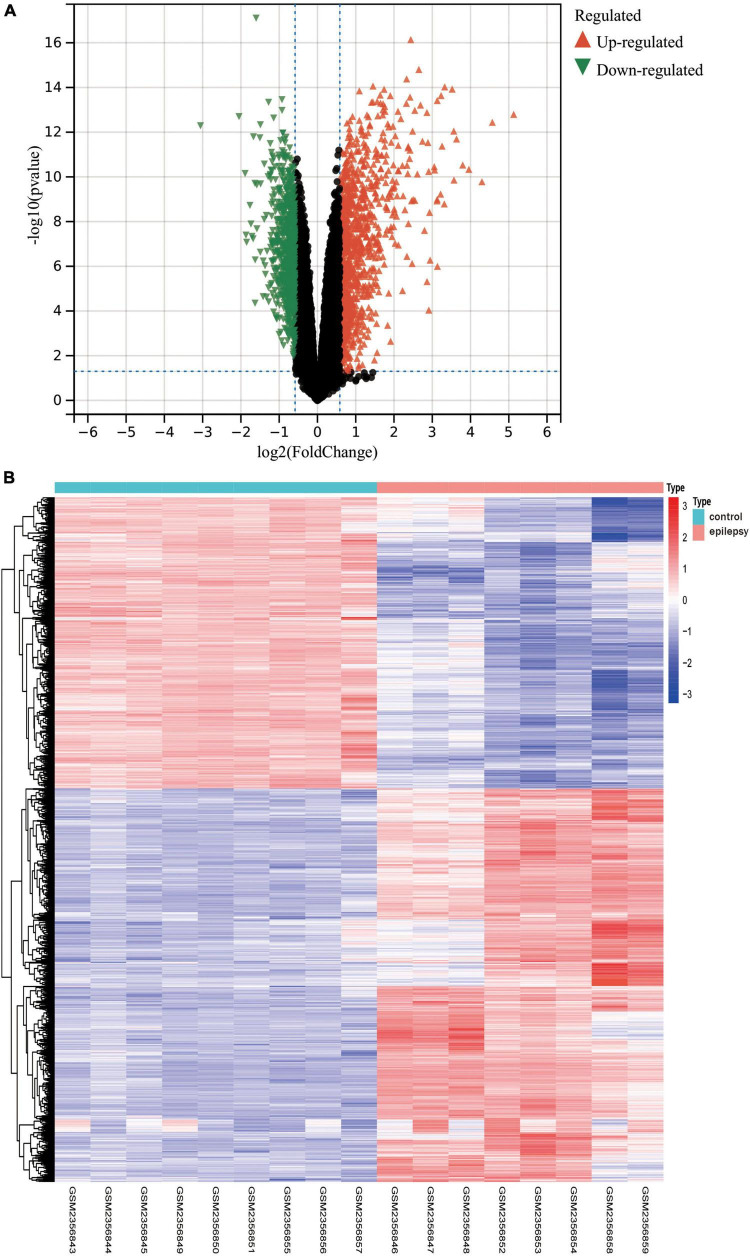
**(A)** The results of the differential expression analysis are shown in the volcano plot. Where the *x*-axis represents log_2_ (ploidy change) and the *y*-axis represents –log_10_ (adjusted *p*-value). Green triangles represent down-regulated genes, red triangles represent up-regulated genes, and black dots represent genes with no significant differential expression. **(B)** Heatmap of the top 50 differentially expressed genes. Each column in the graph represents a sample, each row represents a gene, and the expression status of genes from high to low is represented by red to blue, respectively, at the top of the heat map, blue/red represents the control group/epilepsy group, respectively.

In light of the transcriptome results, we screened 1,800 differentially expressed genes (Supplementary Data Sheet 2), encompassing 765 downregulated genes and 1,035 upregulated genes, after performing differential expression analysis on the dataset GSE88992. The volcano plot (Figure 4A) and heat map display the status of genes with differential expression (Figure 4B).

### 3.4. Construction of WGCNA and determination of core essential modules

First, we ran the Pearson correlation matrix and the average linkage method on all gene pairs. Then, for further modular clustering operations, we utilized the following formula to build a weighted adjacency matrix based on the power function.


$$X\,\mathrm{mn}={\left|Y\,\mathrm{mn}\right|}^{\beta }$$

**FIGURE 5:**
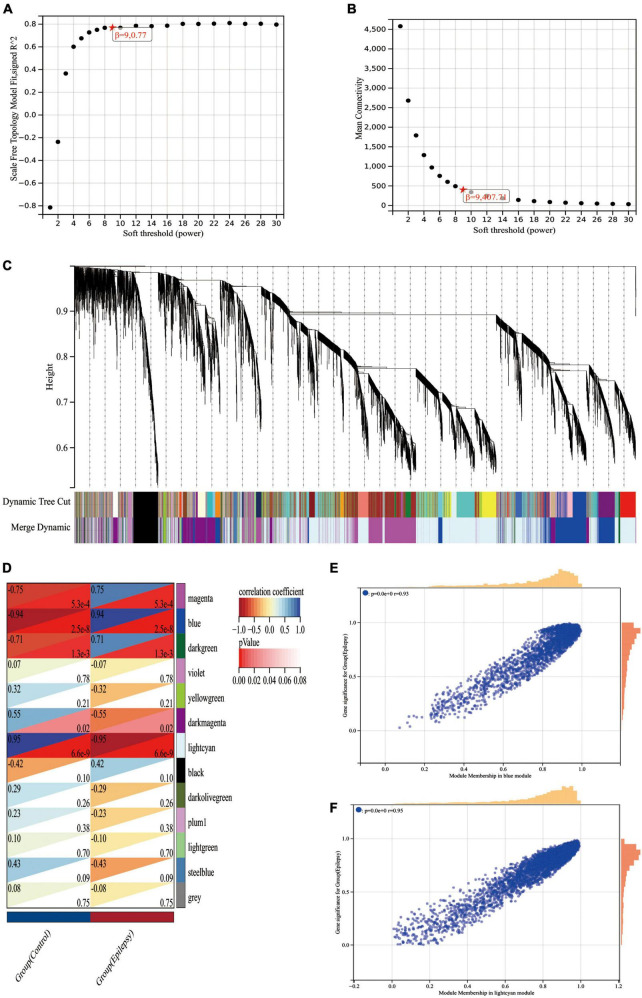
WGCNA analysis of the epilepsy dataset GSE88992. **(A)** Scale-free indices used to analyze the potency of various soft thresholds. Horizontal coordinates represent powers of soft thresholds, and the best soft threshold is marked with an asterisk. **(B)** Average connectivity of various soft thresholds. **(C)** Identification of co-expressed gene modules. A dendrogram of all differentially expressed genes is clustered based on a measure of gene similarity. Cut lines identifying modules are indicated by different colors for each module. **(D)** Heat map of module-clinical phenotype correlation. Each row represents a module; each column represents a clinical feature. Each cell indicates the correlation between the module and the clinical phenotype. The corresponding cor and *p*-values are marked blue and lightcyan modules have the strongest correlation with epilepsy. **(E)** Correlation between module members (MM) and gene significance (GS) in blue modules. r indicates the absolute correlation coefficient between GS and MM. **(F)** Correlation between module members (MM) and gene significance (GS) in lightcyan modules. r indicates the absolute correlation coefficient between GS and MM.

The equation The Pearson correlation coefficient between gene m and gene n is represented by Y mn, while the adjacency between gene m and gene n is represented by X mn. β is a soft threshold parameter that is used in the generation of the weighted adjacency matrix to punish weak associations between genes. In our research, the soft threshold parameter was determined to be 9 (Figures 5A, B). We then turned this adjacency between genes into a topological overlap matrix (TOM matrix), defining 1-TOM as the difference between genes, which better depicts gene connection and adjacency. The TOM matrix-based difference metric enables the classification of genes with similar expression patterns into the same modules. In the gene tree diagram, we set the minimum number of genes included inside a module at 30. We merged some modules with a similar association by calculating the similarity of module feature genes from which a suitable cut line was identified in the gene module dendrogram (Figure 5C). We were finally able to create 13 main modules that linked well with clinical parameters and were shown in the form of heat maps through the computation (Figure 5D). The gray modules in the heat map indicate that the genes in the module cannot be attributed to any module and should be deleted from the research as a priority. We got two most essential modules, blue and lightcyan modules, based on the correlation heat map of clinical characteristics and modules, where the blue module has the biggest positive association with epilepsy and the lightcyan module has the largest negative correlation with epilepsy. To brief, we calculated the correlations between modules and clinical features using two techniques. Method I: Pearson correlation coefficients were determined between ME of modules and clinical characteristics initially, and then modules that were substantially correlated with clinical traits (*p* < 0.05) were found. Method 2: First, the Pearson correlation coefficient [gene significance (GS)] was determined between the expression level of each gene and each clinical characteristic; next, the mean absolute value GS of all genes in the module was obtained. The higher the association between the module and the clinical characteristic, the greater the mean absolute value GS.

Lastly, we generated scatter plots illustrating the GS and MM correlations for each module (Figures 5E, F). When Figures 5D–F are linked, we can find that the blue module (*cor* = 0.94, *p* = 2.5e-8) and the lightcyan module (*cor* = −0.95, *p* = 6.6e-9) had the strongest correlation with the epilepsy group, with a total of 3,315 genes collected from these two modules (Supplementary Data Sheet 3).

### 3.5. Transcriptome differential gene screening

**FIGURE 6:**
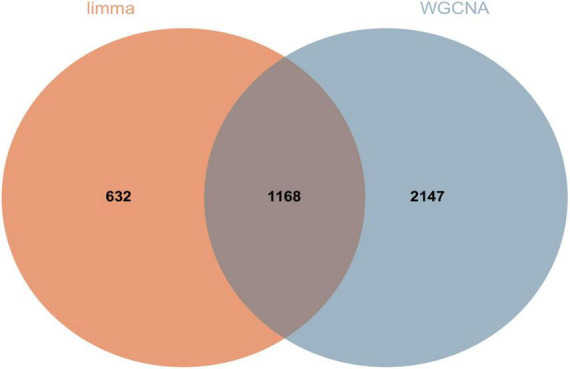
A total of 1,168 DEGs were screened for further analysis. Orange represents 1,800 genes obtained by limma package analysis and blue represents 3,315 genes obtained by WGCNA analysis.

We screened DEGs using two algorithms, with 3,315 genes acquired from WGCNA (1,180 genes in the blue module and 2,135 genes in the lightcyan module) and 1,800 genes obtained through limma package analysis. The genes identified by the two algorithms were then overlapped to provide 1,168 DEGs, which were regarded as transcriptome DEGs. The findings are represented by a Venn diagram (Figure 6) and are documented in a table (Supplementary Data Sheet 4).

### 3.6. Enrichment analysis of DEGs and DEPs

**FIGURE 7:**
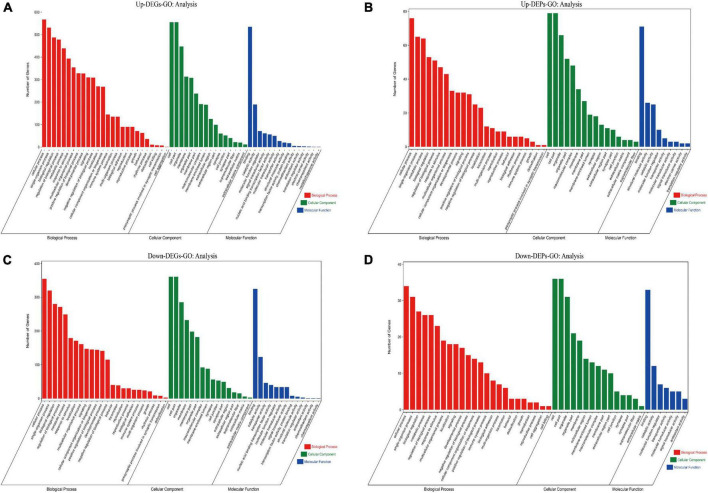
Results of GO enrichment analysis for up/down-regulated DEGs and up/down-regulated DEPs are shown. **(A)** GO enrichment analysis for up-regulated DEGs. **(B)** GO enrichment analysis for up-regulated DEPs. **(C)** GO enrichment analysis for down-regulated DEGs. **(D)** GO enrichment analysis for down-regulated DEPs. The red module shows Biological Process results from GO enrichment; the green module shows Cellular Component results from GO enrichment; the blue module shows Molecular Function results from GO enrichment. The top 25 clusters for Biological Process, Cellular Component, and Molecular Function are selected and displayed, respectively, according to the Number of Genes ranking. DEGs, differentially expressed genes; DEPs, differentially expressed proteins; GO, gene ontology.

To identify the functions of these differentially expressed genes and proteins, we performed GO and KEGG enrichment analyses on the screened up-regulated DEPs and up-regulated DEGs, and down-regulated DEPs and down-regulated DEGs, respectively. We found that both up-regulated DEGs (Figure 7A) and up-regulated DEPs (Figure 7B) were mainly enriched in cellular process, single-organism process, synapse, cell junction, catalytic activity, and molecular function regulator; down-regulated DEGs (Figure 7C) and down-regulated DEPs (Figure 7D) were also enriched in cellular process, biological regulation, synapse, cell junction, catalytic activity, and molecular function regulator. The GO enrichment (Supplementary Data Sheet 5) results indicate that both up/down-regulated DEGs and up/down-regulated DEPs play a role in protein synthesis and synaptic structural integrity.

**FIGURE 8:**
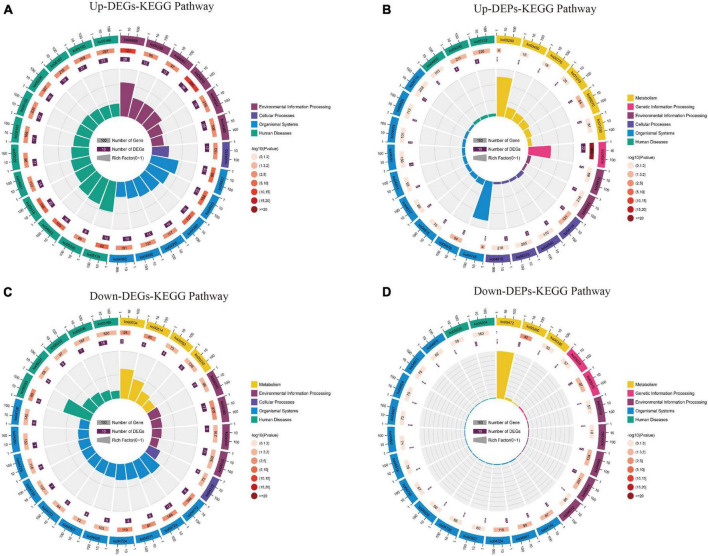
The results of the KEGG pathway analysis for up/down-regulated DEGs and up/down-regulated DEPs are shown as circle plots. **(A)** KEGG pathway analysis for up-regulated DEGs. **(B)** KEGG pathway analysis for up-regulated DEPs. **(C)** KEGG pathway analysis for down-regulated DEGs. **(D)** KEGG pathway analysis for down-regulated DEPs. For enrichment analysis of circle plots, the GO id (or pathway id) label of the first circle corresponds to the “id” of the result data (Supplementary Data Sheet 6) and the “class” of the result data corresponds to the color of the grouping. The bar length of the second circle corresponds to the “bg_num” of the result data, i.e., the number of background genes, while the shade of color corresponds to the *P*-value (or *Q*-value). The third circle corresponds to “fg_num” of the resultant data, i.e., the number of foreground genes. The fourth circle (polarity bar) shows the Rich factor, obtained by dividing by fg_num and bg_num, and corresponds to the data in the ratio column of the “Supplementary Data Sheet 6.” DEGs, differentially expressed genes; DEPs, differentially expressed proteins; KEGG, Kyoto Encyclopedia of Genes and Genomes.

The KEGG pathway (Supplementary Data Sheet 6) shows that up-regulated DEGs are mainly involved in the “TNF signaling pathway,” “MAPK signaling pathway,” “IL-17 signaling pathway,” and “Apoptosis” pathway (Figure 8A); In contrast, the up-regulated DEPs are mainly involved in the “Ribosome,” “Valine, leucine and isoleucine biosynthesis,” and “Cysteine and methionine metabolism” pathways (Figure 8B). Down-regulation of DEGs was mainly involved in “Glutamatergic synapse,” “Dopaminergic synapse,” “cAMP signaling pathway,” and “GABAergic synapse” pathway (Figure 8C); while down-regulated DEPs were mainly involved in “Cell adhesion molecules,” “IL-17 signaling pathway,” and “Glycine, serine and threonine metabolism” pathway (Figure 8D). The KEGG results suggest that both up/down-regulated DEGs and up/down-regulated DEPs play a role in neuroinflammatory, amino acid metabolism, and synaptic function pathways.

### 3.7. Screening for co-expression of differential genes (proteins)

**FIGURE 9:**
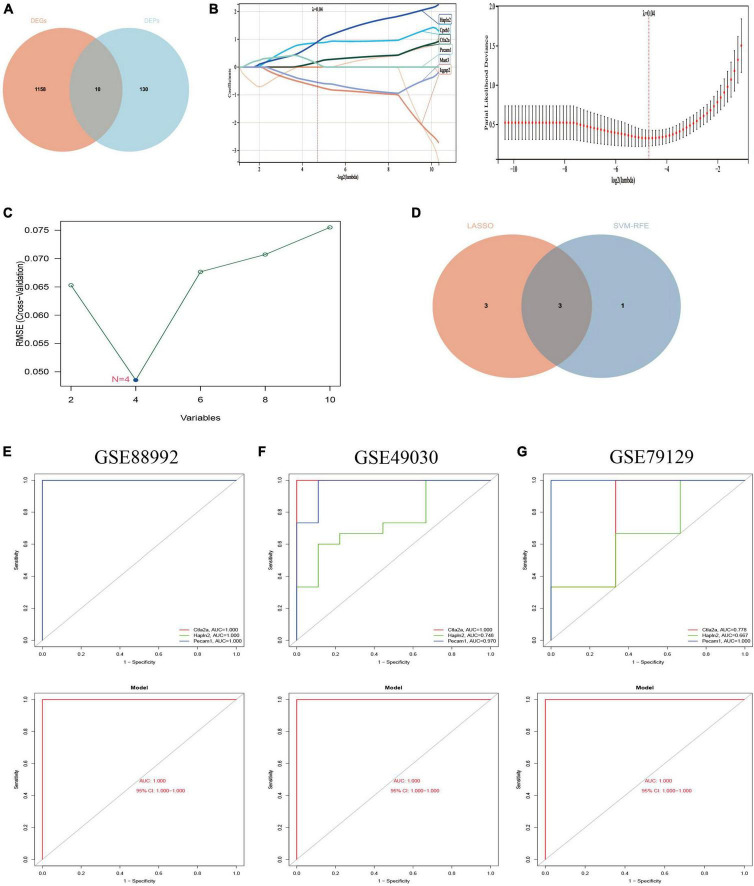
Screening of Hub genes and construction and validation of diagnostic models. **(A)** A total of 10 co-expressed differential genes (proteins) were screened for the next step of the analysis. Red represents 1,168 differential genes (DEGs) obtained by transcriptomic analysis and blue represents 140 differential proteins obtained by proteomic analysis. **(B)** Hub genes were screened using the least absolute shrinkage and selection operator (LASSO) logistic regression method. **(C)** Selection of hub genes by support vector machine recursive feature elimination (SVM-RFE) algorithm of feature selection. **(D)** Venn diagram showing the three hub genes common to the LASSO and SVM-RFE algorithms (Supplementary Data Sheet 8). **(E)** ROC curves for the hub gene (top) and model (bottom) of the training set GSE88992 dataset. **(F)** ROC curves for the hub gene (top) and model (bottom) of the validation set GSE49030 dataset. **(G)** ROC curves for the validation set GSE79129 dataset Hub gene (top) and model (bottom). Different color lines in ROC curves represent different genes.

We overlapped the 1,168 DEGs obtained from the above analysis with the 140 DEPs obtained from the proteomic differential analysis, and a total of 10 co-expressed differential genes/proteins (genes that changed significantly at both transcriptome and proteome levels). The findings are visualized by a Venn diagram (Figure 9A) and documented in the table (Supplementary Data Sheet 7).

### 3.8. Screening of Hub genes and construction and validation of diagnostic models

The potential hub genes in the acute phase of TLE were filtered through the use of two different algorithms. The LASSO regression algorithm was used to narrow down the 10 co-expressed differential genes (proteins) mentioned above, and the 6 co-expressed differential genes most closely related to TLE were identified (Figure 9B). The SVM-RFE algorithm was used to identify a subset of four features from the ten co-expressed differential genes (proteins) listed above (Figure 9C). Consequently, three main characteristic genes (Ctla2a, Hapln2, and Pecam1) were identified as hub genes between these two algorithms (Figure 9D). We utilized the logistic regression algorithm to build a novel diagnostic model based on the three Hub genes and determined the AUC scores of the Hub genes and the model (Figure 9E): Ctla2a (AUC = 1.000), Hapln2 (AUC = 1.000), Pecam1 (AUC = 1.000), and the model (AUC = 1.000). The findings demonstrated that the novel diagnostic model built from the three Hub genes could differentiate between epilepsy patients and healthy control samples.

Furthermore, two independent datasets, GSE49030 and GSE79129, were chosen to validate the diagnostic model’s accuracy. Using the same manner, the AUC scores of Hub genes and models were computed. Ctla2a (AUC = 1.000), Hapln2 (AUC = 0.748), Pecam1 (AUC = 0.970), and the model (AUC = 1.000) were reported in the GSE49030 dataset (Figure 9F). Ctla2a (AUC = 0.778), Hapln2 (AUC = 0.667), Pecam1 (AUC = 1.000), and model (AUC = 1.000) in the GSE79129 dataset (Figure 9G). In conclusion, the diagnostic model we constructed can distinguish epileptic patients from normal individuals and is beneficial for TLE diagnosis and screening during the acute phase.

## 4. Discussion

In this study, we obtained differential proteins (DEPs) and differential genes (DEGs) in the acute phase of TLE by proteomics and transcriptomics, respectively, and performed GO and KEGG enrichment analyses on up/down-regulated DGEs and up/down-regulated DEPs, respectively. Pathway enrichment analysis showed that DEGs and DEPs were mainly involved in neuroinflammation, amino acid metabolism, and synaptic functional pathways. This suggests that TLE and neuroinflammatory responses are closely linked. Previous studies have confirmed the role of neuroinflammation response-related pathways (Mukherjee et al., 2020; Terrone et al., 2020) in the development of TLE and it is worth exploring further.

Following that, the LASSO and SVM-RFE algorithms were applied to identify the following Hub genes, Ctla2a, Hapln2, and Pecam1. Among these three hub genes, Pecam1 was significantly increased in the acute phase of the kainic acid mice epilepsy model and was considered a novel target for epilepsy therapies (Yan et al., 2018). The complete name of Pecam1 is platelet endothelial cell adhesion molecule 1 (also known as CD31 antigen), a transmembrane glycoprotein encoded by the PECAM1 gene on chromosome 17q23.3 (Newman, 1997). It is involved in leukocyte-endothelial interactions and leukocyte transendothelial migration during inflammation. Several studies have shown that patients with multiple sclerosis have significantly elevated expression of Pecam1 in serum and cerebrospinal fluid due to neuroinflammation and disruption of the blood cerebral barrier, and Pecam1 serum concentrations can be used as an indicator of multiple sclerosis blood-brain barrier disruption and a reliable marker of disease activity (Niezgoda and Losy, 2002). A study of ischemic stroke also suggests that Pecam1 may be involved in the extent of early ischemic brain injury mediated by inflammatory responses (Zaremba and Losy, 2002). According to the two studies, Pecam1 gene upregulation in the brain tissue of individuals with acute TLE is likely to cause neuroinflammation and blood-brain barrier disruption, which ultimately contribute to the disease’s progression in the acute phase of TLE. This increases the possibility that Pecam1 gene upregulation could be employed as a biomarker for TLE in acute-phase screening. Ctla2a is cytotoxic T lymphocyte-associated protein 2α, originally identified as being expressed in activating T and mast cells in mice (Denizot et al., 1989). Ctla2a resembles the cysteine protease pre-region structurally (Yamamoto et al., 2002). A major member of the family of lysosomal cysteine proteases is cathepsin L; therefore, Ctla2a demonstrated selective inhibition of cathepsin L (Kurata et al., 2003). In research on ischemic stroke, it was indicated that inhibition of cathepsin L expression in astrocytes contributes to neuronal protection (Xu et al., 2014). Additionally, previous research has revealed that astrocyte activation and inflammation contribute a significant part to the development of epilepsy (Devinsky et al., 2013). The above two studies both suggest that Ctla2a may rescue neuronal death in epileptic lesions by inhibiting the expression of cathepsin L produced by astrocyte activation in neuroinflammation, and thus. In one of the other studies, Ctla2a suppressed the proliferation of endothelial cells in models of experimental choroidal neovascularization and ocular inflammation (Maruyama et al., 2021). In the brains of epileptic patients, blood-brain barrier dysfunction promotes seizures, and sustained seizure activity exacerbates blood-brain barrier damage and angiogenesis, further promoting recurrent seizures (Marchi and Lerner-Natoli, 2013). In conclusion, the pathophysiology of the acute phase of TLE may be suppressed by Ctla2a. On the one hand, it may protect neurons from apoptosis and thus reduce seizures by inhibiting the release of cathepsin L in TLE lesions, and on the other hand, by decreasing endothelial cell growth, it may prevent angiogenesis, lessen the blood-brain barrier damage caused by inflammation in epileptic lesions, and help stop recurring seizures from progressing to chronic seizures. Hapln2, a hyaluronan and proteoglycan link protein 2, also known as brain-derived connexin 1 (Bral1), is essential for neuronal conductivity and extracellular matrix (ECM) formation and has been identified to be involved in a variety of neurological disease pathologies, such as schizophrenia (Martins-de-Souza et al., 2009) and Alzheimer’s disease (Minjarez et al., 2013). Previous work has shown that deletion of Hapln2 decreases the expression of ECM-related proteins (Billingsley et al., 2018). In contrast, an essential part in neuronal death, neuroinflammation, blood-brain barrier disruption, the development of perineuronal networks, and synaptic plasticity-induced epileptic damage is played by the extracellular structural scaffold known as ECM (Pitkänen and Sutula, 2002; Pitkänen et al., 2014; Gorter et al., 2015; Sacks et al., 2018). Thus, Hapln2 regulates the development of epilepsy by affecting the expression of ECM-related proteins. Nevertheless, the Ctla2a and Hapln2 genes have not been adequately studied in epilepsy, and further research is required to determine their possible significance to epilepsy. We next utilized the logistic regression algorithm to build a novel epilepsy diagnosis model incorporating the three hub genes mentioned above. We verified the model’s accuracy utilizing three available public sets of data. Our research’s most remarkable innovation is the use of proteomics, transcriptomics, and machine learning algorithms in the field of epilepsy, with promising results. LASSO and SVM-RFE algorithms are emerging high-accuracy mechanical learned algorithms that have been adopted extensively in many domains, though of course, their usefulness in the medical domain is precisely the same. The LASSO linkage SVM-RFE algorithm has been widely used in the study of clinical diseases, such as using LASSO linkage SVM-RFE to identify biomarkers in patients with acute myocardial infarction to find potential targets for treatment (Zhao et al., 2020) and constructing a new stem cell-related classifier to predict the prognostic of hepatocellular carcinoma patients (Chen et al., 2022), as well as to forecast the evolution for atherosclerotic plaques (Yang et al., 2022) with good results. The combined proteomics and transcriptomics analysis are now also commonly used in the field of epilepsy. Several academics have identified key regulators involved in epilepsy-induced cardiac injury *via* combined proteomics and transcriptomics analysis (Sharma et al., 2021). Nevertheless, there is no research to apply this combination of both in the field of epilepsy (Shan et al., 2021). Thus, the epilepsy diagnosis model developed by integrated proteomics and transcriptomics analysis using LASSO and SVM-RFE algorithms is a daring endeavor and a good supplement to existing diagnostic approaches. Meanwhile, our study reveals epilepsy vulnerability genes that might be participating in the regulating neuroinflammatory response of the pathway. It is our wish that their essential worth will be mirrored in forthcoming research.

Nonetheless, there were certain limits to our research. First, the experimental data of proteomics and transcriptomics were from different experimental platforms and there were some errors. The results obtained from association analysis still need to be validated in experimental studies to confirm our conclusions. Second, the data samples used in this study were all brain tissue samples, lacking the validation of blood or cerebrospinal fluid samples, and the sample size was relatively inadequate, which warranted an additional sample amount and further research and optimization. Third, the applicability of our conclusions to people with epilepsy is uncertain owing to a dearth of relevant data on epileptic individuals.

## 5. Conclusion

We used joint proteomics and transcriptomics analysis combined with both LASSO and SVM-RFE algorithms to determine the Hub genes connected with the acute phase of TLE onset and construct an epilepsy classification diagnostic model based on a logistic regression algorithm. Finally, we demonstrated its superb categorization properties in three separate data sets. In this study, we complemented the available screening and diagnostic instruments in the acute phase of TLE and revealed epilepsy vulnerability genes that might be participating in the regulation of neuroinflammation; it also provided a new perspective to better find drug targets.

## Data availability statement

The mass spectrometry proteomics data have been deposited to the ProteomeXchange Consortium *via* the PRIDE partner repository with the dataset identifier PXD040967 (http://www.ebi.ac.uk/pride).

## Ethics statement

The animal study was reviewed and approved by the Ethics Committee of the Second Affiliated Hospital of Harbin Medical University.

## Author contributions

CH and ZY completed the design of this study and wrote the manuscript. ZL, JL, and YL completed the construction of the epilepsy model used in this study as well as protein extraction and mass spectrometry analysis. XL and CP provided the suggestions for data analysis. YH critically revised the manuscript and provided the suggestions for data analysis. JS provided the constructive suggestions for this study. All authors contributed to the article and approved the submitted version.

## Footnotes

1
https://www.maxquant.org

2
https://www.ncbi.nlm.nih.gov/geo/

3
https://www.perl.org/

4
https://www.kegg.jp/kegg/rest/keggapi.html
